# The Effect of Zero-Valent Iron Nanoparticles (nZVI) on Bacteriophages

**DOI:** 10.3390/v14050867

**Published:** 2022-04-22

**Authors:** Sada Raza, Michał Folga, Marcin Łoś, Zenon Foltynowicz, Jan Paczesny

**Affiliations:** 1 Institute of Physical Chemistry, Polish Academy of Sciences, Kasprzaka 44/52, 01-224 Warsaw, Poland; sraza@ichf.edu.pl (S.R.); michal.folga99@gmail.com (M.F.); 2 Department of Molecular Genetics of Bacteria, Faculty of Biology, University of Gdańsk, Wita Stwosza 59, 80-308 Gdańsk, Poland; mlos@biotech.ug.gda.pl; 3 Phage Consultants, Partyzantów 10/18, 80-254 Gdańsk, Poland; 4 Department of Non-Food Products Quality and Packaging Development, Institute of Quality Science, Poznań University of Economics and Business, Al. Niepodległości 10, 61-875 Poznań, Poland; zenon.foltynowicz@gmail.com

**Keywords:** bacteriophage inactivation, disinfection, antiviral, iron-based nanoparticles, nZVI

## Abstract

Bacteriophages are viruses that attack and usually kill bacteria. Their appearance in the industrial facilities using bacteria to produce active compounds (e.g., drugs, food, cosmetics, etc.) causes considerable financial losses. Instances of bacteriophage resistance towards disinfectants and decontamination procedures (such as thermal inactivation and photocatalysis) have been reported. There is a pressing need to explore new ways of phage inactivation that are environmentally neutral, inexpensive, and more efficient. Here, we study the effect of zero-valent iron nanoparticles (nZVI) on four different bacteriophages (T4, T7, MS2, M13). The reduction of plaque-forming units (PFU) per mL varies from greater than 7log to around 0.5log depending on bacteriophages (M13 and T7, respectively). A comparison of the importance of oxidation of nZVI versus the release of Fe^2+^/Fe^3+^ ions is shown. The mechanism of action is proposed in connection to redox reactions, adsorption of virions on nZVI, and the effect of released iron ions. The nZVI constitutes a critical addition to available antiphagents (i.e., anti-bacteriophage agents).

## 1. Introduction

Bacteriophages (phages) are obligate parasites. They occur in all bacterial habitats. Bacteriophages have a make-up to carry out precise functions of host identification, subsequent metabolism requisition, and reproduction [1]. In most cases, the release of progeny virions (viral particles) results in the death of the host cell.

Laboratories and bacteria-based industries often endure contaminations by bacteriophages. These contaminations spread quickly and are difficult to overcome [2]. The authors of [3] reported that 70% of all biotechnology companies encounter such infections, out of which dairy industries face a 1–10% batch product loss. Most fermentation processes also witness pollution by bacteriophages because they are carried out under non-sterile conditions [4]. Bacteriophages may also insert their genome in the host’s genetic material while forming a prophage. This results in slower growth and decreased byproduct-synthesis efficiency [5,6]. A common means of phage-spread is secondary contamination wherein bacteriophages are introduced in the system via contaminated reactants [7].

From the perspective of laboratories and industries, it is crucial to maintain phage-free bacterial cultures. Different methods are employed to assess such contaminations, such as direct plating with indicator bacteria, batch culture enrichment, and a plating method based on Poisson distribution [8]. Several tests confirming the presence of phages in human urine confirmed their interference with microbiological diagnostic tools [9].

However, phages are not easy to eliminate, especially with routine cleaning processes and disinfectants [7]. Basic methods include the right choice of equipment, appropriate process design, and extensive cleaning and sterilization [10]. Several physical and chemical treatments are popularly employed to decontaminate laboratories and biofoundries [4], e.g., photocatalysis, high-pressure treatments (up to 100 MPa) [11], and ultra-pasteurization [12]. Chemical treatments encompass the use of several disinfectants such as benzalkonium chloride, chlorhexidine, hydrogen peroxide, triclosan, polyvinylpyrrolidone-iodine, alkaline detergent mixtures, potassium peroxymonosulfate (e.g., Virkon S, used in laboratories for decontamination), and quaternary ammonium compound-based sanitizers, to name a few [13,14,15]. Currently, new-generation antiphagents (anti-bacteriophage agents) are being developed. Nanoparticles that can adequately serve as antimicrobial agents are, among others, silver, gold, zinc oxide, titanium oxide_,_ and zero-valent iron [16,17]. The effects of Ag, ZnO, TiO_2_, Au, Cu nanoparticles, and fullerenes on bacteriophage inactivation are explored, but the data are scarce [18,19,20,21]. Fe/Ni nanoparticles also show inactivating properties against bacteriophages (f2) when prepared in a 5:1 ratio, but Ni nanoparticles do not have any such ability [22].

One of the approaches to fight phages is based on the chemistry of iron. The iron uptake mechanism of bacteria is often exploited by phages due to the presence of Fe ions in the tail (via Ferrojan horse hypothesis), resulting in cell lysis [23]. Fe ions present in the tails of bacteriophages assist in adsorption to the receptors of the host cells [24]. However, the most common mechanisms of bacteriophage titer reduction by iron-based compounds are (1) adsorption of virions on the surface of nanoparticles [23], (2) redox reactions [25], and (3) the effect of free iron ions [26].

Most iron hydroxide particles such as hematite (α-Fe_2_O_3_), goethite (α-FeOOH), magnetite (Fe_3_O_4_), and amorphous iron(III) hydroxide (Fe(OH)_3_) display similar virus adsorption capacity. However, the rate of adsorption varies in the order FeOOH > Fe_2_O_3_ > Fe_3_O_4_ ≈ Fe(OH)_3_ [27]. Up to 1.5log of MS2 bacteriophages and rotavirus (RV) are removed from samples due to adsorption to hematite nanoparticles within 45 min [28]. Iron oxide amended biosand filters also help disinfection via electrostatic adsorption of negatively charged virion particles to the positively charged iron oxides [29]. Nanoporous iron oxide ceramics also eliminate viral contamination from water using ferroxane nanoparticles [30]. MS2 and φX174 bacteriophages can also be inactivated or irreversibly adsorbed to iron granules in batch reactors [31].

To enhance the inactivation of some bacteriophages by photocatalysis, ferrous sulfate is added to cause a reduction of phage titer by the production of hydroxyl radicals (Fenton’s reaction) [32]. Metal catalyzed Fenton systems cause virus inactivation in natural systems and water treatment by complex oxidation processes [33]. Fenton’s reaction produces strong oxidants (hydroxyl radicals and Fe(IV) (FeO^2+^) ions) by combining hydrogen peroxide and ferrous ions [34]. Fe(II) quickly interacts with the amino acids in the protein capsids of bacteriophages, and the subsequent production of oxidants upon contact with H_2_O_2_ ultimately leads to the degradation of the virus [34]. The effect of [FeO_4_]^2−^ (Fe(VI)) on MS2 phages assumes the Chick–Watson model, where the inactivation rate constant is directly proportional to the concentration of Fe(VI) ions, temperature, and inversely proportional to pH [35].

Free iron ions released from iron-based disinfectants affect bacteriophages. The relation between the concentration of ferrous ions (Fe^2+^) at low doses and bacteriophage inactivation is linear. Higher doses show limited decontamination, probably due to flocculation [26]. Fe^2+^ ions alone (0.1 mM) have been reported to inactivate up to 1.5log of MS2 phages compared to 4log in the case of the Fenton process (a mixture of Fe^2+^ ions with H_2_O_2_) [34]. Fe^2+^ has a more substantial effect on f2 than Fe^3+^, probably owing to the limited solubility of the latter [25,36]. Differences in the titer reduction arise from varying susceptibility to ferrous inactivation in MS2, φ X174, and mammalian viruses. Microbes with a negative charge show a greater affinity towards ferrous ions. Another factor that affects disinfection is the aggregate formation of virions that establishes a protective layer against inactivating agents [37].

Recently, zero-valent iron (ZVI) has been used to fight viruses. ZVI might act via all three described mechanisms, i.e., by adsorption, by redox reactions when the conversion of Fe(0) to Fe(II) and Fe(II) to Fe(III) causes inactivation (e.g., MS2 [38]) and via release of free iron ions. In the case of bacteriophages, the inactivating properties of nano ZVI (nZVI) are oxygen-dependent, as observed in the case of f2 [36]. It is also noteworthy that nZVI is more reactive against f2 phages (5.1log) than nZVI nanoparticles (0.5 log) due to their smaller particle size (hence higher surface reactivity), larger surface area, and ease of dispersion [39]. Apart from MS2 and φ X174 bacteriophages, nZVI-containing columns can also inactivate eukaryotic viruses (e.g., Aichi virus) [40]. Tulane virus (TV) and murine virus (MNV) can also be removed from water by a mixture of nZVI and sand in the treatment columns. Bacterial cellulose loaded with nZVI enhanced the inactivation efficiency of MS2 phages. Interestingly, this inactivation was caused by O^2−^ and Fe(IV) radicals within 5 min of interaction [41]. Moreover, TV-inoculated lettuce also decreased PFU when washed with nZVI water [42].

Certain oxidants such as KMnO_4_, H_2_O_2_ can enhance the reactivity of nZVI in water remediation and CrO_4_^2−^ to overcome surface passivation, which is often followed by nZVI reduction [43]. Methods of improving the reactivity of nZVI include metal doping to produce bimetallic nanoparticles with Cu, Ni, or Ag that increase the dissolution of iron [44].

In this paper, we explore the effect of nZVI on four bacteriophages (T4, T7, MS2, M13) and establish how various phages behave under the influence of those nZVI nanoparticles. The nZVI nanoparticles were synthesized in an unusual way, without the use of FeSO4, as described in the cited literature, in order to facilitate the separation of anions from the post-reaction mixture [45]. The nZVI nanoparticles, primarily acting as oxygen scavengers, were prepared using chlorides as starting materials. The nZVI was synthesized by reducing iron (III) chloride with sodium borohydride in an aqueous solution. The process was carried out in a glass flask with a capacity of 3 dm^3^ equipped with a gas inlet and outlet. The synthesis parameters have been specially established and are protected by patents [46].

## 2. Materials and Methods

### 2.1. Chemicals

Liquid LB medium constituting 10 g/L of NaCl, 10 g/L of tryptone, and 5 g/L of yeast extract. LB-Agar used to prepare plates was prepared as LB medium, but with the addition of 15 g/L of agar. LB medium was used to prepare overnight and refreshed cultures. Both LB-Agar and LB pre-mix were ordered directly from Carl Roth, Schoemperlenstraße, Germany. TM Buffer (pH 7.4) was composed of 10 mM Tris, 10 mM MgSO_4_, and deionized water and supplemented with 5 μM CaCl_2_. All the media were sterilized before use.

### 2.2. Consumables

Plasticware purchased from Bionovo (Legnica, Poland): sterile falcon tubes (NeoCulture centrifuge tubes, made of PP—50 mL, self-standing), Eppendorf-type tubes—1.5 mL (B-1429 and B-2278), which were proved not to adsorb virions [47].

### 2.3. Synthesis of nZVI

The nZVI was synthesized by reducing iron (III) chloride with sodium borohydride in an aqueous solution. The process was carried out in a glass flask with a capacity of 3 dm^3^ equipped with a gas inlet and outlet. The synthesis parameters have been specially established and are protected by patents [46].

Typical reaction procedure: 1 L of 0.05 M orange-colored iron (III) chloride was poured into the flask, the assembly was purged with argon, and 1 L of 0.15 M aqueous sodium borohydride solution was added dropwise at a rate of 0.25–0.3 mL/s until no further black precipitate was noticed and no hydrogen evolution. The reaction mixture was left for 24 h, then an additional 200 mL of the borohydride solution was added in portions and left for 24 h to stabilize the sludge. The precipitate after decantation was washed with distilled water until the Cl^-^ ions were completely removed. The precipitate was then filtered through a 20 DEN nylon fabric filter (app. 420 Mesh) and dried by lyophilization. The obtained nZVI was stored in a sealed container under a protective atmosphere of argon for further use. Three forms of nZVI were used, namely pristine (reduced) ZVI, partially oxidized (PO ZVI), and oxidized ZVI (O ZVI). PO ZVI was obtained by exposing ZVI to air for 2 min and O ZVI for two days. The main difference was that ZVI samples were kept in the inert atmosphere of argon during experiments, whereas PO ZVI and O ZVI were exposed to air throughout the experiments.

### 2.4. Bacteriophages

To prepare phage lysates, early logarithmic cultures of Escherichia coli BL21 or E. coli C3000 were infected. Once the lysis was complete, phages were precipitated with polyethylene glycol. This precipitate was centrifuged and diluted with 1M NaCl. Purification was carried out by ultracentrifugation of bacteriophages in a CsCl gradient. Ultimately, the suspension was dialyzed against TM buffer, and 0.2 μg/mL of viscolase (A&A Biotechnology, Gdańsk, Poland) was added to digest the released DNA and reduce the overall viscosity. MS2 phages were directly filtered through 0.22 μm syringe filters without precipitation and centrifugation steps. TM buffer was purged by bubbling argon through it for 40 min.

### 2.5. Bacteria

Stock plates were used to pick a single colony of E. coli BL21 or E. coli C3000 (the strains were obtained from the Institute of Biochemistry and Biophysics, PAS, Warszawa, Poland). This colony was then transferred to 10 mL of LB medium to prepare the bacterial cultures. This sample was incubated overnight at 37 °C in the shaker (Orbital Shaker-Incubator ES-20, 200 rpm). The next day, the culture was refreshed by mixing 2.5 mL of the overnight culture with 7.5 mL of LB medium and incubating at 37 °C for approximately 1 h.

### 2.6. Double Overlay Titration for Phage Analysis

Petri plates were plated with 20 mL of LB-agar medium and allowed to solidify. Once dried, 4 mL of top agar (LB medium containing 0.5% agar) along with 200 μL of the refreshed culture of E. coli were poured on the top of it. After preparing appropriate dilutions of the phage solution, eight droplets of 5 μL solution were spotted onto the top agar layer. The number of plaques was counted after a 24 h incubation of the plates at 37 °C. All the experiments were performed in triplicates. Student’s *t*-test was performed to evaluate the statistical significance. * *p* < 0.05; ** *p* < 0.01; *** *p* < 0.001.

### 2.7. Dose-Response Experiments

ZVI nanoparticles (powder) were dissolved in argon-purged TM buffer in an inert atmosphere (argon) in Schlenk flasks. The transfer was carried out using a glove box. The Schlenk flask was evacuated, purged with argon thrice, and used to prepare the suspensions. Concentrations of 10 mg/mL, 1 mg/mL 0.1 mg/mL and 0.01 mg/mL of nZVI were studied to understand the effect of concentration on phage titer. Controls were prepared with TM buffer and degassed TM buffer. To each sample, 500 μL of 10^8^ PFU/mL of phages were mixed with TM buffer or ZVI suspension (of the calculated concentration) to make up the volume to 5 mL. The samples were mixed throughout the experiments. The samples were mixed throughout the experiments at 300 rpm using an orbital shaker.

An hourly experiment was run, wherein the phages were titrated every hour for 6 h. In the case of nZVI, the experiment was performed without changing the inert atmosphere of the samples (using septa and syringes). PO ZVI and O ZVI were exposed to air. The number of viable virions was evaluated using the double overlay method.

### 2.8. Experiments with Fe (II) and Fe (III) Salts

Solutions of iron (II) chloride tetrahydrate (Merck, Darmstadt, Germany) and iron (III) chloride hexahydrate (Merck, Darmstadt, Germany) were prepared in an inert atmosphere. The experiment proceeded in the same manner as for ZVI (see above). The plaques were counted the next day after overnight incubation at 37 °C for 24 h.

### 2.9. SEM, STEM, and TEM Analysis

Scanning electron microscopy (SEM) and scanning transmission electron microscopy (STEM) images were obtained using an FEI Nova NanoSEM 450 scanning electron microscope. The samples of reduced ZVI nanoparticles were prepared inside a glove box and transferred to SEM quickly for visualization. Oxidized ZVI nanoparticles were prepared in contact with air. Powder nZVI was deposited onto carbon conductive and adhesive tape in both cases. The unbounded nZVI was shaken off the sample.

TEM study was conducted using an electron microscope Tecnai Spirit BioTWIN with a digital camera. The samples were prepared for negative staining by placement on C400Cu100 grids (400 Mesh, copper, and carbon layer coating) manufactured by EM Resolutions. Samples were negatively stained with 2% uranyl acetate in water at room temperature for 15 s.

## 3. Results

### 3.1. Characterization of ZVI Nanoparticles

**Figure 1:**
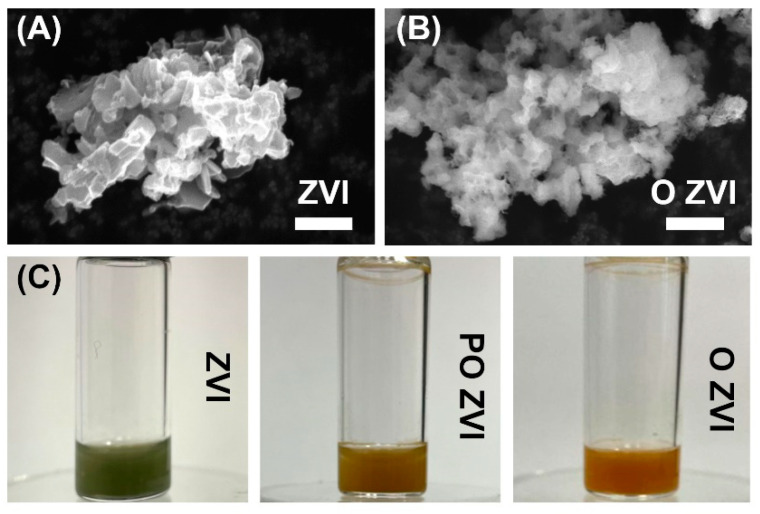
SEM pictures of (**A**) pristine (reduced) ZVI and (**B**) oxidized ZVI (O ZVI). Scale bars correspond to 1 µm. (**C**) Visual discrimination between ZVI, partially oxidized ZVI (PO ZVI), and oxidized ZVI (O ZVI).

ZVI nanoparticles were analyzed by scanning electron microscopy (SEM). A comparison between reduced (pristine) and oxidized ZVI (O ZVI) is shown in Figure 1. Large aggregates were visible because powdered samples were used for sample preparation and not ZVI dispersed in the solvent. This was done to speed up the analysis and avoid unwanted oxidation of pristine ZVI. The sub-100 nm size of ZVI obtained by us was proved previously using SEM [45] and via Sherrer’s equation [48]. Both pristine and oxidized ZVI nanoparticles were similar in size and shape, but oxidation changed the surface features of the studied material. The surface of oxidized ZVI resembled macroscopic rust, as expected.

### 3.2. Influence of nZVI on Bacteriophages

An hourly analysis was carried out to inspect the effect of ZVI nanoparticles on bacteriophages. Three states of ZVI were tested, namely: ZVI in its native, reduced form, partially oxidized (PO ZVI), and fully oxidized (O ZVI). To obtain partially oxidized samples (PO ZVI), the particle suspensions were exposed to air just before starting the experiment (exactly 2 min). Completely oxidized samples were prepared by exposing the nZVI suspension to air for two days. The difference in the appearance of ZVI, PO ZVI, and O ZVI is shown in Figure 1C. Three forms of ZVI were chosen to verify which mechanism of action (i.e., adsorption, redox reaction, or effect of released ions) is dominant in the case of phages. Samples exposed to ZVI were kept in an inert atmosphere for the whole experiment, and ZVI did not change its appearance throughout the experiment, i.e., it remained green. PO ZVI was exposed to air, and it changed its color from green to orange during the experiment. O ZVI was exposed to air, and it remained orange throughout the experiment.

Four phages were selected for the experiment, namely, T4, T7, M13, and MS2. It is established that 96% of all known phages belong to the order *Caudovirales*, or tailed phages (including T4 and T7) [49]. MS2 bacteriophage was chosen as it is popularly used as a eukaryotic virus surrogate [50,51]. MS2 is a non-enveloped phage, and it is a positive sense single-stranded RNA virus like SARS-CoV-2. MS2 is used as a SARS-CoV-2 surrogate for COVID protection study [52]. *Inoviridae*, comprising, among others, M13, f1, and fd is the most broadly investigated order as nanocarriers [53]. Filamentous bacteriophages find wide applications as antitumor agents utilizing phage display techniques [54]. They are also readily genetically engineered using well-established cloning methods and are highly immunogenic [53].

No significant increase in inactivation was observed with increasing ZVI concentration from 1 mg/mL to 10 mg/mL, probably due to aggregation. Moreover, there was no visible effect for concentration as low as 0.01 mg/mL.

**Figure 2:**
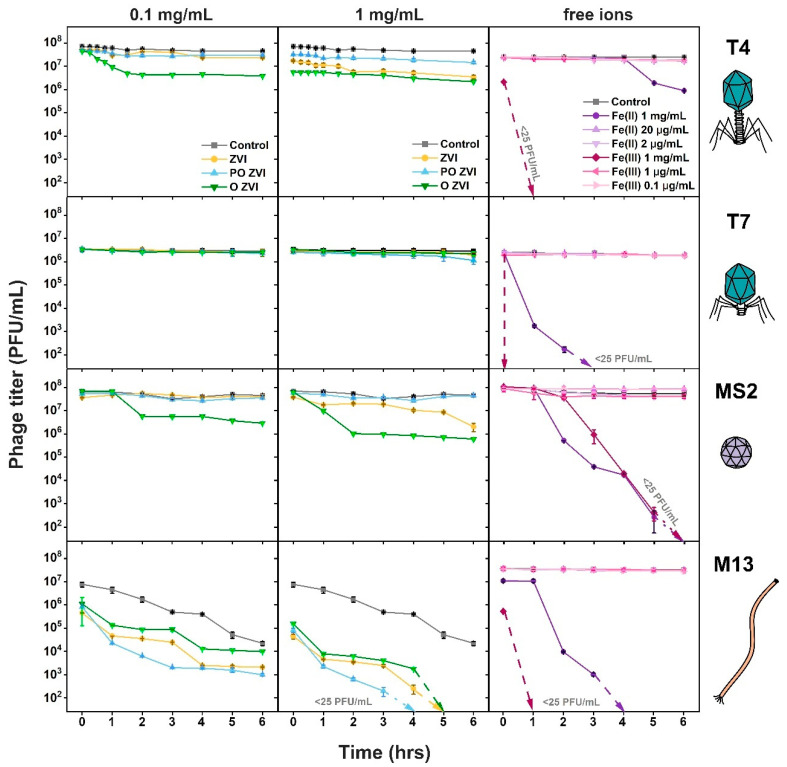
PFU/mL calculation of bacteriophages in TM buffer, ZVI (0.1 and 1 mg/mL), partially oxidized and M13 in the presence of Fe (II) (1 mg/mL 20 μg/mL and 2 μg/mL) and Fe (III) ions (1 mg/mL, 1 μg/mL and 0.1 μg/mL). Cartoons depicting phages are reprinted from [55] with permission from Elsevier.

The inactivating effect of ZVI on the selected phages was in the order M13 > MS2 ≈ T4 > T7, with reductions of 7log, 2log, 1.5log, and 0.5log in the PFU/mL within 6 h, respectively (Figure 2). It is noteworthy that PO ZVI is most effective against M13, while T4 and MS2 are inactivated to greater extents by O ZVI. T7 is most resistant against ZVI nanoparticles in all forms. The decrease in control M13 samples was due to the mechanical agitation applied to overcome sedimentation of ZVI.

**Figure 3:**
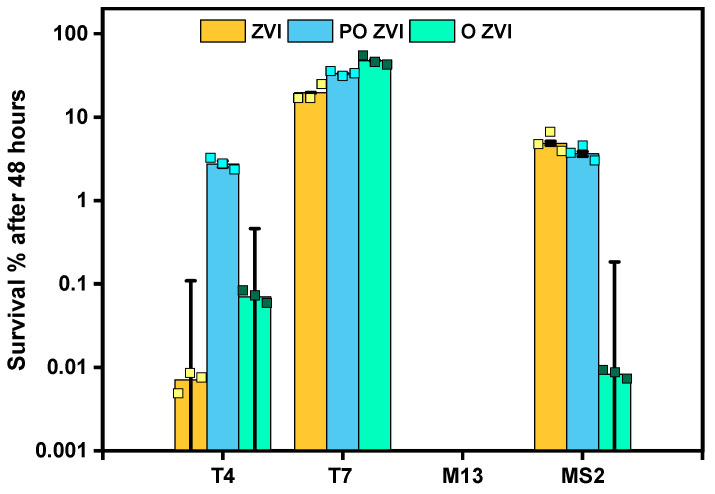
48 h experiment of bacteriophages in the presence of ZVI, PO ZVI, and O ZVI, all at a concentration of 1 mg/mL. The squares on each bar represent the three biological repetitions and the bar itself is the average. The initial concentration of the phages was around 10^7^ PFU/mL in all cases. *p* values regarding control samples were smaller than 0.001 (***) in all cases.

Next, phages were incubated with all three forms of ZVI for 48 h. ZVI did not change its color even after the experiment, as it was kept in an inert atmosphere. The percentages of survived phages (normalized by the initial concentration of each phage sample) are shown in Figure 3.

M13 did not survive 48 h of exposure to any studied nZVI form. The detection limit in this experiment was around 25 PFU/mL, which corresponds to around 7 log inactivation.

On the contrary, T7 was barely affected, with almost no reduction after 6 h. 48 h incubation caused only around 80.4%, 66.6%, and 52.3% reduction upon exposure to ZVI, PO ZVI, and O ZVI, respectively.

In the case of MS2 and T4, O ZVI showed the highest reduction of titers, i.e., between 99.99% and 99.92% inactivation. Prolonged exposure (48 h) caused a PFU/mL decrease of 5log and 4log in MS2 and T4, respectively.

Prolonged incubation significantly improved the efficacy of pristine ZVI against T4 from around 1log to around 5log titer reduction. This was not the case against MS2, which showed a similar 1–2 log reduction after 6 h and 48 h.

PO ZVI, which showed very little activity against MS2 and T4 within the first 6 h, reached only around 2log reduction after 48 h. The magnitude of inactivation of MS2 by ZVI and PO ZVI was similar and much smaller than O ZVI.

### 3.3. Influence of Fe(II)/Fe(III) Ions on Bacteriophages

To evaluate the influence of free Fe^2+^ and Fe^3+^ ions, phages were exposed to iron (II) chloride and iron (III) chloride solutions. First, we tested the scenario in which all ZVI (i.e., 1 mg/mL) is dissolved, resulting in a 1 mg/mL concentration of iron ions. Furthermore, to understand the effect of the more realistic concentrations, we calculated an approximate equivalent concentration of released ions and titrated bacteriophages against 20 μg/mL or 2 μg/mL for Fe^2+^, and 1 μg/mL or 0.1 μg/mL for Fe^3+^. To calculate an approximate amount of Fe^2+^ ions in a solution of ZVI (1 mg/mL), we referred to the available data on the release of ions upon dissolution of magnetite in water. At pH 7, 250 μM Fe^2+^ ions (i.e., 139 μg/mL) were released from a solution of Fe_3_O_4_ (0.7 mg/mL) [56]. The European Chemicals Agency states that dissolved iron concentration in 10 mg/mL of Fe_2_O_3_ is less than 1 μg/mL. For estimations, we also used published data on the solubility of iron compounds in water [57].

The realistic concentrations of Fe^2+^ and Fe^3+^ ions did not affect the studied phages within 6 h of incubation (Figure 2). Higher concentrations of Fe^2+^ and Fe^3+^ ions were taken as controls to analyze their impact on phages, if any. We found that 1 mg/mL of Fe^2+^ and Fe^3+^ ions caused complete removal of T7, MS2, and M13 within 6 h. Fe^3+^ fully removed T4, but the titer was only slightly affected by Fe^2+^ ions. The action of Fe^3+^ against T4, M13, and T7 is much quicker (T7 removed after around 30 s of exposure) than MS2.

### 3.4. Electron Microscopy Visualization of Phages Exposed to ZVI

**Figure 4:**
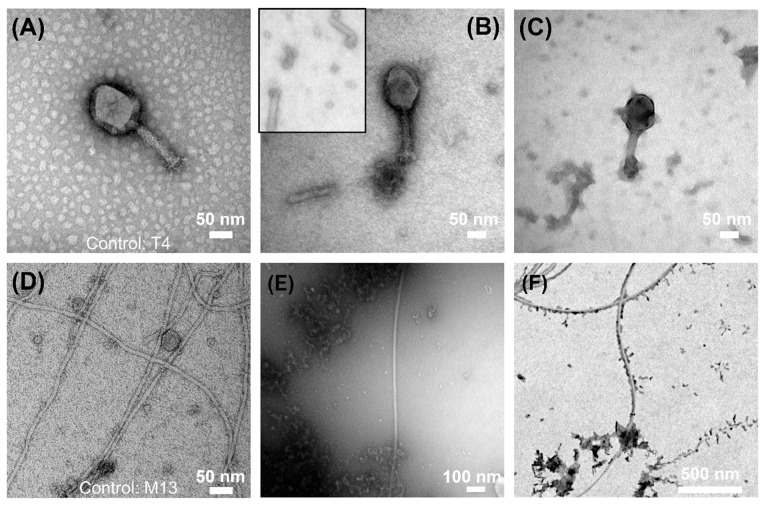
TEM visualization of bacteriophages. (**A**) T4 bacteriophages in the absence of zero-valent iron. (**B**,**C**) T4 bacteriophages exposed to O ZVI. Inset in (**B**) shows the detached tails, which were in abundance in the sample. (**D**) M13 bacteriophages in the absence of zero-valent iron, (**E**) Supernatant of the suspension containing only a small number of M13 virions, among which majority were in contact with nZVI particles. (**F**) STEM image of the freshly agitated suspension wherein nZVI and M13 appeared in close proximity.

To visualize the morphological changes caused by ZVI, we executed TEM analysis. We focused on PO ZVI and O ZVI for experimental reasons (ZVI oxidized during sample preparation). We chose T4 and M13 bacteriophages (unexposed virions are shown in Figure 4A,D, respectively), as the effect was most pronounced in these cases. T4 phages exposed to O ZVI were damaged with numerous stems detached from the virions (Figure 4B) and capsids losing integrity (Figure 4C). Upon exposition of M13 to ZVI, very few virions were present compared to control, despite the same titers in both samples. We hypothesize that the nanoparticles get adsorbed to the virions causing sedimentation. Therefore, only a small number of virions was present in the supernatant. Most of these virions were found only in close proximity to nZVI (Figure 4E). To prove that most virions were present in the sediment, we visualized agitated samples. As opposed to the supernatant, numerous virions were visible in freshly suspended samples. Again, M13 virions were in contact with nZVI (Figure 4F). We performed three biological repetitions of the experiment, and visualized each sample thrice. At least 7 pictures were taken in random places of each sample.

## 4. Discussion

Three forms of ZVI were chosen to verify which mechanism of action (i.e., adsorption, redox reaction, or effect of released ions) is dominant in the case of phages. Pristine ZVI is capable of redox reactions with viral components, even in the degassed medium and inert atmosphere. In the case of partially oxidized ZVI, the nanoparticles are undergoing redox reactions in the sample due to the exposure to air. Moreover, release of iron ions is also possible in this case, creating local environments capable of phage inactivation. Oxidized ZVI can release iron ions, but no redox reaction occurred because the nanoparticles are completely oxidized. In all cases adsorption of virion at the surface of ZVI particles is possible. This is also a requirement for redox reactions in pristine ZVI.

Several findings required analysis: (1) M13 is very vulnerable to all studied forms of ZVI, whereas T7 appears almost completely resistant; (2) T4 and T7 belong to the same family (*Caudovirales*), yet significant inactivation of T4 is observed; (3) Pristine ZVI is active against M13 and T4, but not T7 and MS2; (4) In the case of T4, PO ZVI shows much smaller activity comparing to ZVI and O ZVI. To rationalize obtained results, we analyzed them in the context of properties of studied phage virions (Table 1).

The observed effect of ZVI on bacteriophages might arise from three mechanisms: (a) redox processes, (b) the effect of released Fe^2+^ and Fe^3+^ ions on bacteriophages, and (c) the adsorption of phages onto ZVI nanoparticles. We designed the experiment to conclude which mechanism prevails in the case of ZVI. From Figure 2, it can be concluded that ions’ effect is less significant than other mechanisms since no reduction in titer was observed with realistic concentrations of Fe^2+^ and Fe^3+^ ions. However, the effect of ions in the local, confined environment in the proximity of ZVI upon adsorption of virions onto ZVI nanoparticles can be present. In such circumstances, the concentration of ions is higher than in bulk, causing the inactivation of pre-adsorbed virions. Similar reasoning is also valid for active products of redox reactions. Redox reactions involving capsid proteins are possible only in the proximity of the ZVI surface.

All viruses showed no inactivation upon the addition of realistic amounts of ions. Therefore, it is likely that ions released from ZVI into the bulk solution did not cause the inactivation of non-adsorbed virions. Thus, we assumed that all observed reduction was a consequence of collisions [71] or adsorption [47] of virions at the surface of ZVI. This did not exclude the possibility of T7 adsorbing at ZVI but still surviving. Experiments at high concentrations of iron ions showed that T7 was more susceptible than T4 (Figure 2). Because inactivation of T4 by ZVI and O ZVI was substantial, and T7 was almost unaffected (Figure 2 and Figure 3), it suggested that T7 did not adsorb at the surface of any studied form of ZVI. It was shown by Armanious et al. that the adsorption properties vary even for similar phages (MS2, fr, GA, and Qβ). Authors linked the adsorption characteristics to differences in their capsid surface properties [64].

M13 phages were most vulnerable to all studied forms of ZVI, with PO ZVI being slightly more potent than ZVI and O ZVI. In addition, free ions (Fe^2+^ and Fe^3+^) significantly influenced M13 titers. Little to no data are at hand regarding the interaction of filamentous bacteriophages with zero-valent iron nanoparticles. It is unclear which factor triggered the inactivation in our experiments. We hypothesized that this was due to contact of virions with ZVI nanoparticles, as the dynamics of the titer decrease were similar in all ZVI, PO ZVI, and O ZVI (Figure 2). It became more evident with TEM pictures (Figure 4E) when the number of virions decreased in the presence of ZVI nanoparticles. We showed that nZVI attached to M13 virions and caused sedimentation resulting in a subsequent decrease in observed titer (Figure 4F). We hypothesized two probable causes of phage titer reduction when M13 was in contact with ZVI: (*i*) the phages were structurally damaged, or (*ii*) the adsorption of nanoparticles to the filaments blocked attachment to pilus or hampered the ability of the capsomeres to get dissolved in the bacterial cell membrane. STEM pictures (Figure 4F) suggested that M13 bacteriophages did not lose their filament shape. We also performed an experiment with Tween 20 to carry out phage recovery since Tween 20 has a higher affinity for surfaces. No viable virions were detected. Moreover, a lack of zone of inhibition around the sprayed ZVI nanoparticles indicate the inactivity of the adsorbed M13 phages.

The next question was why T4 phages were vulnerable to reduced ZVI (up to 4log decrease when exposed to O ZVI for 48 h), and T7 was not. Both phages belong to the same family (*Caudovirales*) but differ significantly in structure. First, T4 has a long (around 90 nm) contractile tail, with long (145 nm) fibers at the end [60,61]. The tail of T7 is short (around 20 nm) and non-contractile, while fibers are attached close to the capsid [63]. Secondly, T4 has a base plate [60], and T7 does not [62,63]. The base plate coordinates host recognition or other environmental signals with sheath contraction [72]. TEM observations (Figure 4) revealed a significant number of detached tails, suggesting that the connection of the tail with the head was targeted. This is supported by our measurements of the zeta potential of nZVI (from around −5 mV to around −2 mV), which are in line with the literature data (cf. Figure 2 in [73]). ZVI with a negatively charged zeta potential interacted with positively charged parts of virions, i.e., tail and fibers. Oxidation generates positively charged iron ions in the proximity of the surface. Thus, this mechanism was most important for pristine ZVI samples, where no or very little oxidation occurred (green color did not change during the experiment (Figure 1c)). This is also in line with results on MS2, which was not affected by ZVI to the same extent as T4, as MS2 does not have a tail. Tail detachment is also observed when phages are incubated with sodium hydroxide [15]. Alternatively, the damage of phage morphology can be caused due to the attachment of the phage head to heavier molecules, i.e., nZVI. Richter et al. showed positively charged nanoparticles to attach to negatively charged parts of virions, e.g., capsid and neck of T4, causing efficient deactivation [74].

O ZVI was effective against MS2 and T4 to a similar extent. This is most likely due to the action of released ions on the adsorbed virions. This is in line with the negative values of the zeta potential of both phages (Table 1). It was found that microbes with a negative charge show a greater affinity towards ferrous ions [37]. As positive ions are released from nZVI (Fe^2+^ and Fe^3+^) upon oxidation, the electrostatic binding between a locally positive surface and the positively charged tails and fibers would be unlikely. According to this explanation, tailed phages (in our case, T7 and T4) should be least affected by oxidized ZVI while getting inactivated by pristine ZVI. Moreover, phages such as MS2 that completely lack a positively charged structural part are most affected by oxidized ZVI. A T4 titer drop was observed but due to the other reasons, namely structural damages.

A potential hypothesis is that two distinct mechanisms of action, namely (1) interaction between ZVI (negative zeta potential) and positively charged tails of T4, and (2) effect of positively charged ions released from O ZVI on virion having overall negative zeta potential (MS2 and T4), also explained the smaller effect of PO ZVI. In this case, the number of positive ions is large enough to screen the interactions with the positively charged tail of T4 but not large enough to cause inactivation via the action of these ions on negatively charged capsid.

## 5. Conclusions

The anti-bacteriophage activity of zero-valent iron varies strongly against different phages. M13 appears most vulnerable, with up to 7log inactivation. On the other hand, T7 is barely affected, with less than 1log inactivation after 48 h of incubation with the relatively high concentration of ZVI nanoparticles. We postulate that this is a consequence of different adsorption characteristics and that only virions close to zero-valent nanoparticles are destabilized. Interestingly, studies on nZVI against MS2 bacteriophages show a variety of results ranging from minimum inactivation (deaerated conditions) [38], to moderate inactivation (our results, Figure 3) to complete inactivation (7log) [75].

We also show differences in the activity of various forms of ZVI, namely pristine (reduced) ZVI, PO ZVI (ZVI nanoparticles that are exposed to air and oxidize while incubated with phages), and O ZVI (oxidized ZVI, which was completely oxidized before the addition of phages). We hypothesized that ZVI affects the phages via redox reactions involving virion proteins, PO ZVI via reactive species generated upon oxidation of ZVI with oxygen that dissolves in the liquid during the experiment, and O ZVI via released iron ions and not redox as it is already fully oxidized at the beginning of the experiment. When M13 is excluded from the analysis, PO ZVI appears least potent, and O ZVI is most potent against bacteriophages. Pristine ZVI shows moderate activity against T4 but not MS2. This is likely due to the interaction between nanoparticles with negative zeta potential with the T4 tail, which bears a positive charge.

ZVI nanoparticles are currently being used to prepare filters to obtain virus-free water samples. Advancements in this field are required to achieve a better phage inactivation.

## Figures and Tables

**Table 1:** Comparison of physical characteristics of MS2, M13, T4, and T7 bacteriophages.

	MS2	M13	T4	T7
**shape**	icosahedral	filamentous	myophage (long contractile tail)	podophage (short tail)
**size**	23 to 28 nm [58]	880 × 5.5 nm [59]	115 × 85 nm capsid, 92 × 24 nm tail, 145 nm motile fibers [60,61]	60 nm capsid [62], 23 nm tail [63], fibers usually bound to capsid, with 90° kink separating two parts around 30 nm and around 20 nm long [63]
**genetic material**	ssRNA	ssDNA	dsDNA	dsDNA
**genome size**	3569 nucleotides	6407 nucleotides	168 903 bp	39 936 bp
**zeta** **potential at pH around 7**	around −40 mV [64]	−18 mV [65]	−26 mV [47]	−21.1mV [66] −5.49 mV [67]
**dipole moment**	NA	NA	24 kD (200 kD when the fibers are extended) [68,69]	5096 D [70]. The charges are unequal, with a larger negative charge on the head and a smaller charge on fibers/little tail [66].

## Data Availability

Data are available from the corresponding author upon reasonable request.
